# Supplementary material for: Complexome profiling of the Chlamydomonas psb28 mutant reveals TEF5 as an early PSII assembly factor
Source: Plant Cell. 2025 Mar 17;37(6):koaf055. doi: 10.1093/plcell/koaf055 (PMC12164590; doi:10.1093/plcell/koaf055)
Supplement: koaf055_Supplementary_Data [file koaf055_supplementary_data.zip › psb28_interactive.zip!/plots/intensity/qualityPlots/peptides/ATPD_Cre11.g467569.t1.1.html]

### Cre11.g467569.t1.1

Trivial name: ATPD  
SeqCoverage [median]: 53.90  
Mass: 24.00 kDa  
  
 

| Sequence | MissedCleavage | Mass [Da] | wtIntensity | muIntensity | Score |
| --- | --- | --- | --- | --- | --- |
| ALVELADEK | 0 | 986.53 | 205860.54 | 146444.15 | 176.44 |
| ALVELADEKGK | 1 | 1171.64 | 42982.17 | 25222.08 | 120.96 |
| ALVELADEKGKLEAVHADVDAVAGLMK | 2 | 2791.48 | 95826.83 | 80800.70 | 147.11 |
| ALVELADEKGKLEAVHADVDAVAGLMKENAK | 3 | 3233.70 | 155020.69 | 176398.07 | 161.01 |
| EAGFQQYTINWLNLLVEK | 0 | 2165.12 | 1333.68 | 0.00 | 69.11 |
| EAGFQQYTINWLNLLVEKDR | 1 | 2436.24 | 1008.28 | 0.00 | 219.87 |
| GKLEAVHADVDAVAGLMK | 1 | 1822.96 | 38478.67 | 36616.39 | 188.04 |
| GKLEAVHADVDAVAGLMKENAK | 2 | 2265.18 | 55663.61 | 47475.48 | 171.84 |
| KLQELTGSK | 1 | 1002.57 | 306497.44 | 257138.70 | 209.03 |
| KLQELTGSKNIK | 2 | 1357.79 | 0.00 | 1387.73 | 67.46 |
| KNEVSESYAK | 1 | 1153.56 | 14755.92 | 7184.51 | 141.08 |
| LEAVHADVDAVAGLMK | 0 | 1637.84 | 5630.33 | 9933.66 | 132.05 |
| LEAVHADVDAVAGLMKENAK | 1 | 2080.06 | 6525.55 | 6803.53 | 100.92 |
| LEQEQQFLIAK | 0 | 1345.72 | 211853.44 | 241686.56 | 287.77 |
| LEQEQQFLIAKK | 1 | 1473.82 | 0.00 | 5409.99 | 105.17 |
| LQELTGSK | 0 | 874.48 | 272888.44 | 172363.21 | 156.35 |
| LQELTGSKNIK | 1 | 1229.70 | 0.00 | 714.58 | 44.30 |
| LSALIMNPVVESDK | 0 | 1514.80 | 184490.99 | 212214.05 | 293.80 |
| LSALIMNPVVESDKK | 1 | 1642.90 | 58618.49 | 113679.04 | 198.87 |
| LSALIMNPVVESDKKR | 2 | 1799.00 | 403695.22 | 424062.00 | 199.68 |
| NEVSESYAK | 0 | 1025.47 | 25613.89 | 17306.73 | 145.17 |
| SAVKLEQEQQFLIAK | 1 | 1730.96 | 521321.54 | 928322.62 | 268.90 |
| SAVKLEQEQQFLIAKK | 2 | 1859.05 | 12181.11 | 22880.91 | 147.83 |
| VADQLTK | 0 | 773.43 | 439680.04 | 344596.73 | 188.81 |
| VADQLTKEMTAK | 1 | 1333.69 | 71533.12 | 86915.98 | 124.38 |
| VADQLTKEMTAKLS | 2 | 1533.81 | 1460.19 | 6448.30 | 58.12 |
|  |  |  | 3132920.2 | 3372005.7 |  |
